# Swelling of micro-hydrogels with a crosslinker gradient†
†Electronic supplementary information (ESI) available: A SasView implementation of the described form factor is provided, as well as an exploration towards the properties of the corona of dangling ends. See DOI: 10.1039/c7cp02434g


**DOI:** 10.1039/c7cp02434g

**Published:** 2017-06-06

**Authors:** Niels Boon, Peter Schurtenberger

**Affiliations:** a Division of Physical Chemistry , Department of Chemistry , Lund University , SE-22100 Lund , Sweden . Email: niels.boon@fkem1.lu.se

## Abstract

A heterogeneous distribution of crosslinker in micro-hydrogels (microgels) results in a non-uniform polymer density inside the particles. Identifying the morphology of the hydrogel backbone enables a bottom-up approach towards the structural and rheological properties of microgel systems. On a local level we use a Flory–Rehner inspired model that focuses on highly swollen networks, characterized by a Poisson's ratio of 1/4. Our *ab initio* calculations take account for the nonuniform distribution of crosslinker species during the synthesis of poly(*N*-isopropylacylamide) (PNIPAM) microgels, yet the method is also applicable to other microgel architectures. We recover a single-particle density profile that is in close agreement with SAXS data. Comparison with experimental data confirms that the surface of the cross-linked particle is decorated with dangling polymers ends of considerable size.

## Abstract 2

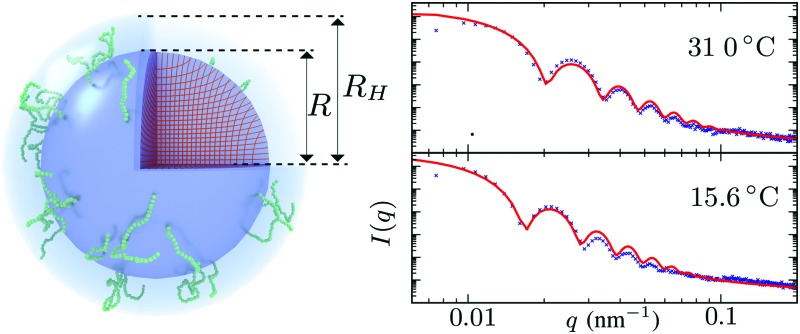
The swollen structure of microgels with a crosslinked-polymer backbone is recovered by considering the crosslinker gradient inside the particles.

## 1 Introduction

Microgels are sub-micron sized particles with a backbone of crosslinked polymer. They offer high control (*in situ*) over shape, size, charge,^1^,^2^ and soft interactions^3^,^4^ by stimuli such as temperature, pH,^5^,^6^ or salt concentration.^7^ Ionic microgels can even change size with the volume fraction of the sample.^8^ Poly(*N*-isopropylacylamide) (PNIPAM) polymers exhibit a temperature-induced volume phase transition from coil^9^ to globule around 33 degrees Celsius^10^ and can be shaped into microgels *via* precipitation polymerization.^11^ This yields particles that reversibly change from a nearly-incompressible sphere to a swollen network upon varying the temperature, which can be used to explore novel routes towards complex structure formation, opens new possibilities for drug delivery^12^–^14^ and cell cultivation,^15^ or can be used to design fluids with adjustable rheological properties.^16^,^17^ The addition of crosslinkers during synthesis is essential for creating stable hydrogel cores and increasing the amount of crosslinkers results in more rigid particles. Crosslinkers get predominantly incorporated early during the particle synthesis process due to a different reactivity of the crosslinker species and the NIPAM monomers. The resulting particle has, therefore, a relatively dense core and a much softer surface region.^18^–^24^ Dispersing the crosslinker more homogeneously throughout the particle can be promoted by a controlled feed of crosslinker into the reaction mixture.^21^,^25^ Nevertheless, many intriguing observations on microgel systems hint at the fact that the inhomogeneity itself is a major contributor to the interesting behaviour that is found for these systems.^23^,^26^,^27^ The precise internal structure of the particles and how this quantitatively affects the properties of the suspension is, however, still unclear.

**Fig. 1:**
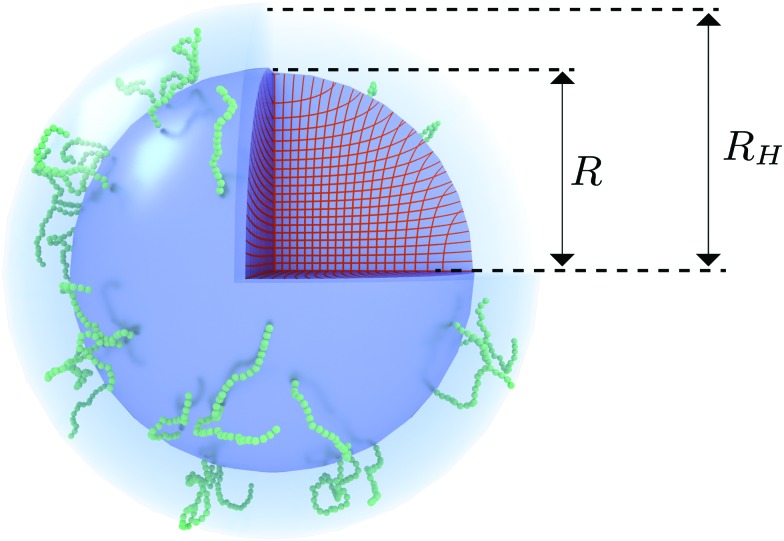
Visualization of the proposed density profile. The (red) grid lines show the actual calculated anisotropic swelling profile of the crosslinked core. The existence of a layer of dangling ends (sketched in green) will attribute to a difference between the (core) radius *R* and the measured hydrodynamic radius *R*_H_.

The celebrated model for describing the swelling of hydrogels is the Flory–Rehner theory,^28^–^30^ which has originally been derived to analyze the elastic properties of rubber-like materials. This model can be used to capture the volume phase transition that is observed for PNIPAM microgels, yet it has been unsuccessful in predicting the elastic moduli and soft interactions between the particles.^24^ Deviations from bulk-gel behavior due to the crosslinker density gradient may be attributed to this. It has been suggested by Fernandes *et al.* that a spatially varying crosslinker distribution can be considered by means of a ‘matryoshka’ approach, *i.e.* by considering the gel particle as a collection of shells with different gel parameters.^31^ Low values of the elastic moduli of the interacting outer shells are achievable in this way. The interactions between the particles consequently follow a rubber-like (Hertzian) interaction potential^3^,^32^ that relates the deformation of the network upon contact to an effective inter-particle force. Although the approach of Fernandes *et al.* points at the importance of accounting for the distribution of crosslinkers, deviations from a freely-swelling gel emerge when the outer shells are able to swell in the radial direction but are constrained in the angular direction due to their attachment to a more-rigid inner core.^33^,^34^ It has been suggested that the very low crosslinker concentration near the surface could also result in coronas of dangling polymer chains with considerable size,^34^ as is shown in Fig. 1. This implies that the pair interactions between the gel particles should rather be described by considering the interaction of the dangling ends, while (at low till moderate densities) the deformation of the underlying network is minimal. Rheology experiments seem to support this assumption.^35^ Interpenetrating dangling ends could enable the system to reach volume fractions beyond close packing^36^ and may explain the longevity of metastable (crystalline) states of PNIPAM suspensions as has been observed experimentally.^37^

In this work we focus on highly swollen gels only, which enables simple scaling relations of the swelling ratio w.r.t. parameters such as the crosslinker density. We demonstrate how this approach is supported by measurements of the Poisson ratio for bulk PNIPAM gels. For hydrogel particles with inhomogeneous crosslinking the swelling can be calculated using a bottom-up analysis towards the distribution of crosslinkers during synthesis. This approach yields a form factor that agrees with intensity curves from scattering experiments. A significant difference between the fitted radius and the measured hydrodynamic radius indicates the presence of a voluminous layer of dangling ends that extends up to 25% beyond the gel-core radius. This study therefore could help to elucidate the nature of soft interactions between microgels, which is crucial for understanding both static and dynamic properties of microgels systems.^24^,^32^,^38^

## 2 Theory and model

### 2.1 Homogeneous hydrogels

**Fig. 2:**
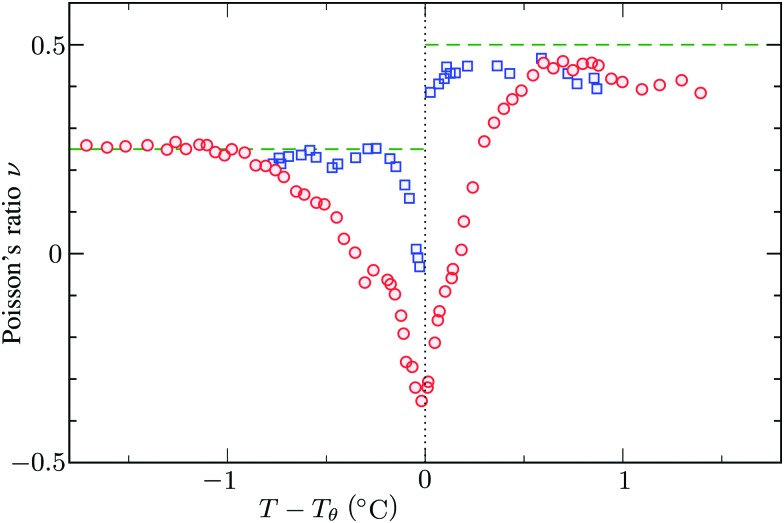
Experimental data for the Poisson's ratio *ν* of two bulk PNIPAM gels with different fractions of crosslinkers, measured by Hirotsu *et al.*^47^ For temperatures below *T*_*θ*_ the network is swollen and the ratio *ν* approaches 1/4 as described in the main text. On the other hand, for *T* > *T*_*θ*_ we observe *ν* → 1/2 as the collapsed gel shows a rubber-like (incompressible) strain response. In this plot we used *T*_*θ*_ = 33.8 °C and *T*_*θ*_ = 33.3 °C for the data corresponding to the circles and the squares, respectively.

Swelling of charge-neutral hydrogels is induced by excluded-volume interactions between the segments that constitute the polymer network. For a self-avoiding network of *N* monomers in a volume *V* of sufficiently high polymer concentration, but not high enough to induce saturation effects,^39^ the associated free energy of this excluded-volume interaction is1
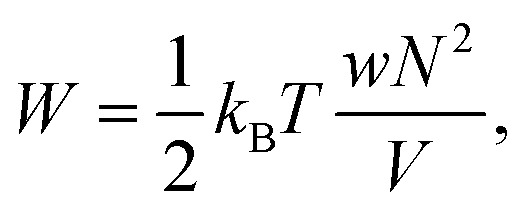
where *w* is the effective excluded volume per monomer. This free energy represents the Flory scaling argument for a single self-avoiding chain. Analogous to the single chain, for which the swelling is balanced by a the entropic cost of chain extension,^30^ the resistance to extension of the gel is driven by the entropic penalty associated with a decrease of microscopic chain configurations. The earliest models for the elasticity of rubbers assumed the crosslinkers to be attached to an affine background with respect to macroscopic deformations of the medium,^30^ and the polymer chains that span between the crosslinkers are modelled with the free energy of Gaussian springs2
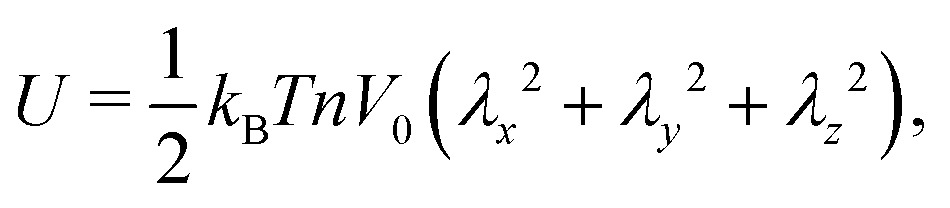
which can be referred to as the elastic energy. In eqn (2), *n* equals the density of chains in the reference state, *i.e. n* = 2*N*_c_/*V*_0_, with *N*_c_ the number of cross linkers in a tetrafunctional network. The *λ*_i_ parameters specify the principal extension ratios of the network with respect to the collapsed phase with volume *V*_0_. In this reference state the average entropic free energy is (3/2)*k*_B_*T* per chain for ideal chains. Note that for isotropic swelling the polymer volume fraction *φ* satisfies *φ* = *φ*_0_/*λ*^3^, *λ*_*x*_ = *λ*_*y*_ = *λ*_*z*_ = *λ*, with *φ*_0_ = *Nw*/*V*_0_ the volume fraction in the reference state. Although this Gaussian relation might break down for chains that approach maximum stretching, in this work we assume that the gel properties is sufficiently stretchable and non-Gaussian behavior is ignored. The affine model has been successfully applied in modelling the elasticity of rubbers,^40^ yet fixing the crosslinker positions ignores their spatial degrees of freedom. This has lead to the introduction of the phantom model,^41^ which focuses on fluctuation of the crosslinkers around a mean position that is affine with the strain. For the tetrafunctional network that we consider this approach results in *n* = *N*_c_/*V*_0_. It has been suggested that polymer networks tend to approach the phantom limit upon significant swelling.^42^ Nevertheless, a recent study also demonstrated the importance for real networks of ‘loop defects’, which are chains that loop back to the same crosslinker and therefore do not contribute to the elasticity. Consequently, in this study we will assume a linear dependence between *n* and the crosslinker density, *n* ∝ *N*_c_/*V*_0_, yet we leave the proportionality factor to be determined experimentally. In equilibrium, the swelling pressure *Π*_ex_ that follows from eqn (1) cancels the elastic pressure *Π*_el_ from eqn (2), *e.g.*3
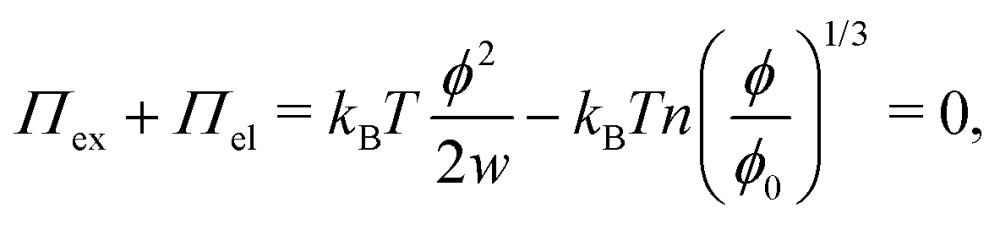
and the low-density (hydrogel) regime of the Flory–Rehner theory,^28^,^29^ *φ* ≪ 1, is recovered. We find *φ*/*φ*_0_ = (2*nw*/*φ*_0_^2^)^3/5^, and the radius of a homogeneous gel particle, therefore, scales as *R* ∝ *N*_c_^–1/5^. It has been shown that this scaling relation also holds for PNIPAM microgel particles with a supposedly heterogeneous crosslinker distribution.^16^,^43^–^45^ This indicates that for these particles the local crosslinker densities scale linearly with the total amount of crosslinker added during synthesis, as we will demonstrate below. Although the swelling pressure and the elastic pressure cancel each other in equilibrium, the elastic moduli probe their strength indirectly.^46^ We proceed by calculating the bulk modulus 
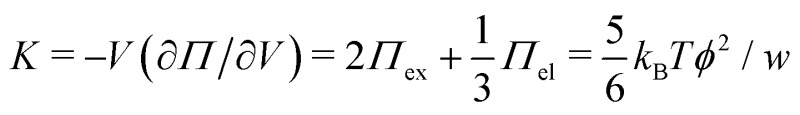
, which can thus be determined if *φ* and *w* are known. Furthermore, the shear modulus describes the additional elastic energy of the network as a result of a shear strain *x* → *x* + *γz*, *i.e. λ*_*z*_^2^ → *λ*^2^(1 + *γ*^2^), and satisfies *G* = (∂*U*(*γ*)/∂*γ*)/(*γV*) = *k*_B_*Tn*(*φ*/*φ*_0_)^1/3^. We find Poisson's ratio, which is the signed ratio of transverse strain to axial strain, is *ν* = (3*K* – 2*G*)/(6*K* + 2*G*) = 1/4. The latter is confirmed by the results of Hirotsu for bulk PNIPAM gels^47^ (sufficiently) below the volume phase transition temperature (VPTT), as can be observed in Fig. 2. On the other hand, for temperatures above VPTT we observe *ν* → 1/2, as the network expels water and becomes incompressible. Unfortunately, the elegant demonstration of these limiting values has not been recognized in ref. 47 itself, as well as some of the later work that refers to this, as the equation for the shear modulus seems to be missing a factor 2 there. We emphasize that equilibrium properties are considered here: on short time scales the initial *ν* is closer to values of 1/2 before the network equilibrates to the external force and some water is expelled or absorbed.^48^

### 2.2 Heterogeneous crosslinking

Microgel synthesis usually takes place at temperatures well above the VPTT. In this regime, the polymer experiences the water as a bad solvent and repels most of its water,^10^ resulting in a simple box-profile for the radial density profile of the growing particle during synthesis. However, the difference in affinity between the monomers and the crosslinker species to attach to the growing cluster results in a non-uniform deposition of the crosslinker within the particle. This growth process typically takes tens of minutes for PNIPAM^49^ and can therefore safely be considered reaction limited. The monomers and the crosslinkers are consumed while the particles with volume 
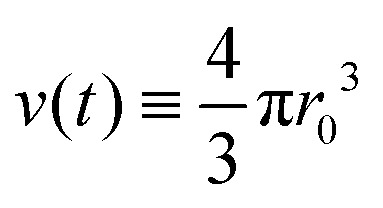
 grow to their final radius *r*_0_ → *R*_0_. By assuming that the crosslinker is a minority species, the conservation of mass implies that at any time *t* the remaining monomer concentration in solution is *c*_m_(*t*) = *c*_m_(0)(1 – *v*(*t*)/*v*(*t* → ∞)). The reaction rates that describe the depletion of monomers and crosslinkers from solution are 
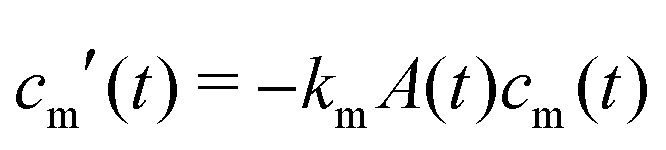
, and 
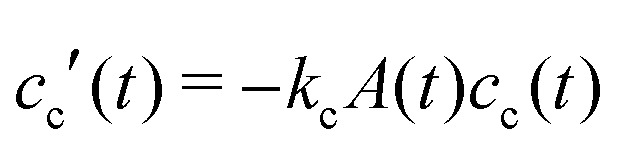
, with *A*(*t*) the number of reactive sites on the particle, *c*_c_(*t*) the crosslinker concentration in solution, and *k*_m_ and *k*_c_ the reaction rate constants for the monomers and the crosslinkers, respectively. One, therefore, finds *c*_c_(*t*)/*c*_c_(0) = (*c*_m_(*t*)/*c*_m_(0))^*k*^, where *k* ≡ *k*_c_/*k*_m_ is the relative reaction rate. The local fraction of crosslinker *ρ*_c_0__(*r*_0_) in the final (collapsed) particle is set by relative deposition ratio of crosslinkers and monomers on a growing particle with radius *r*_0_, *i.e.*
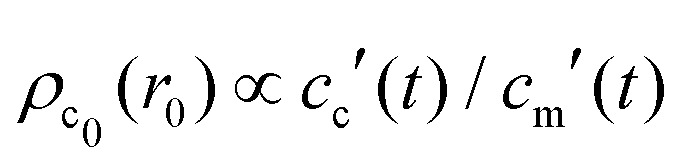
, yielding4
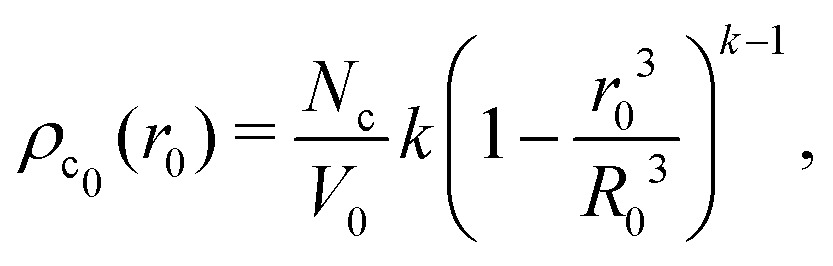
and, thus, confirms that the local crosslinker densities in the collapsed particle scale linearly with *N*_c_, yielding *R* ∝ *N*_c_^–1/5^. Furthermore, Acciario *et al.*^50^ and Wu *et al.*^49^ have reported experimental data suggesting that the relative reaction rate *k* ≈ 2 for PNIPAM microgels that are crosslinked with *N*,*N*′-methylenebisacrylamide (BIS). In the remainder of the text we will therefore set *k* = 2. This doubled probability of the crosslinker species to get incorporated into the growing network can be interpreted as its ability to attach with either of its two distinct reactive groups.

### 2.3 Swelling of heterogeneous microgels

Spherical symmetry allows the heterogeneous density profile of the swollen polymer network to be described by a coordinate mapping *r*_0_ → *r*(*r*_0_) corresponding to the projection of the crosslinker radial coordinates *r*_0_ in the collapsed network to coordinates *r* in the swollen gel. For any *r*(*r*_0_), the local extension ratio of the polymer chains in the radial direction satisfies *λ*_r_(*r*_0_) ≡ ∂*r*(*r*_0_)/∂*r*_0_, while the extension in the two orthogonal angular directions is *λ*_*θ*_(*r*_0_) ≡ *r*(*r*_0_)/*r*_0_. The local volume fraction can be expressed as *φ*(*r*_0_) = *φ*_0_*λ*_r_^–1^(*r*_0_)*λ*_*θ*_^–2^(*r*_0_). A local mean-field approach can be applied to express the elastic and the excluded-volume energy, eqn (1) and (2) as
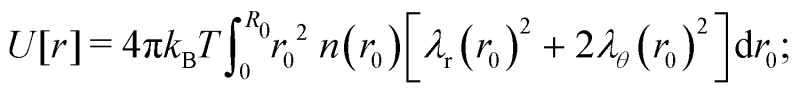

5
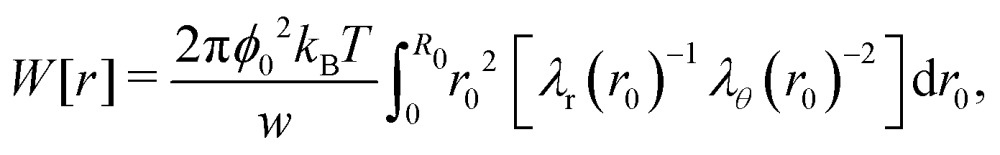
and their sum is minimized for6
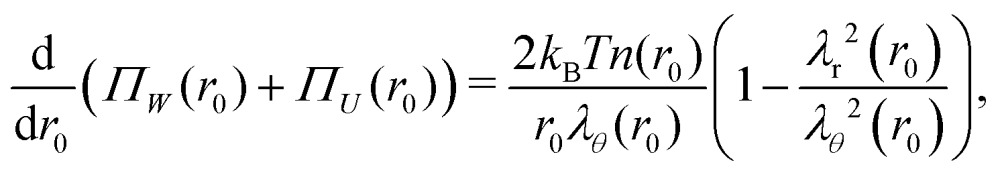
where *Π*_*W*_(*r*_0_) ≡ –*k*_B_*Tn*(*r*_0_)*λ*_r_(*r*_0_)*λ*_*θ*_^–2^(*r*_0_) is the normal radial stress, and the local excluded-volume pressure can be recognized in *Π*_*U*_(*r*_0_) ≡ *k*_B_*T*(*φ*_0_^2^/2*w*)*λ*_r_^–2^(*r*_0_)*λ*_*θ*_^–4^(*r*_0_), analogous to eqn (2) and (1), respectively. Note that *n*(*r*_0_) ∝ *ρ*_c,0_(*r*_0_) resembles the calculated heterogeneous crosslinker distribution in the collapsed particle, while for homogeneous gels eqn (6) would vanish as *λ*_r_ = *λ*_*θ*_ in that case. By definition, *r*(0) = 0 and as there is no net force acting on the gel–water interface, *i.e. Π*_*W*_(*R*_0_) + *Π*_*U*_(*R*_0_) = 0. Eqn (6) and these boundary equations determine *r*(*r*_0_) by means of a second-order differential equation that can be solved numerically. We choose a trial value of the extension ratio in the origin, *λ*_0_ ≡ *λ*_r_(*r*_0_) = *λ*_*θ*_(*r*_0_) and calculate *Π*(0) = *Π*_*W*_(0) + *Π*_*U*_(0). Then, we solve for *r*(*r*_0_) and *Π*(*r*_0_) on a grid, using that *r*(*r*_0_ + Δ*r*_0_) = *λ*_*θ*_(*r*_0_)·Δ*r*_0_, where *λ*_*θ*_(*r*_0_) is calculated from *Π*(*r*_0_) and *r*(*r*_0_). Also the ‘new’ values for *Π*(*r*_0_ + Δ*r*_0_) can be calculated, as these follow directly from eqn (6). This process propagates until *Π*(*R*_0_) is obtained. Should *Π*(*R*_0_) be positive (negative), then a larger (smaller) value of *λ*_0_ is required in the next iteration. Generally, a high-precision solution is found rapidly.

## 3 Results and discussion

### 3.1 Numerical results

**Fig. 3:**
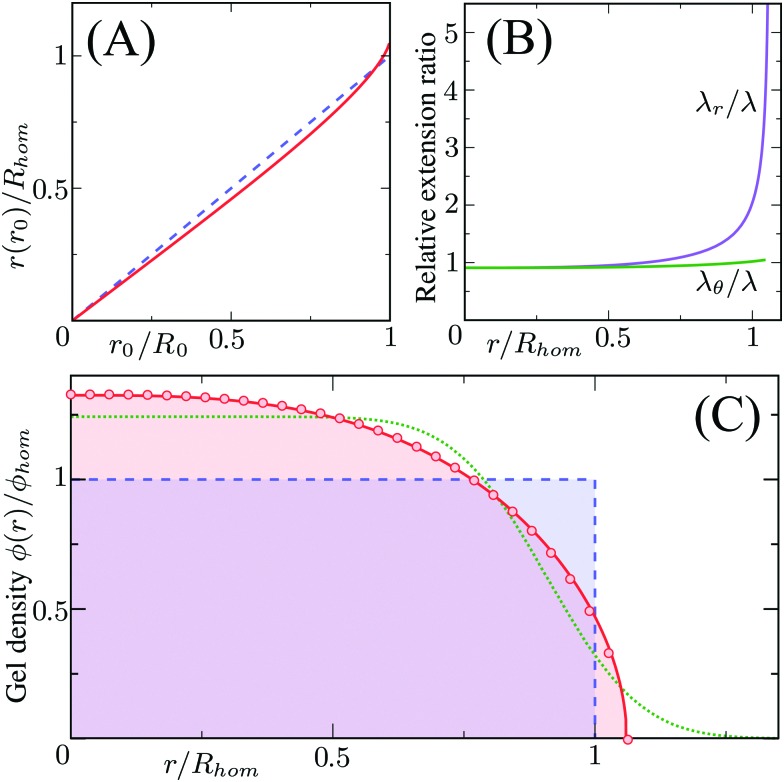
Calculated swelling of the particle, showing in (A) the calculated radial coordinate *r*(*r*_0_) (solid line), normalized to the swollen radius *R*_hom_ of a particle with a homogeneous crosslinker distribution (*k* = 1, indicated by the dashed line). The corresponding relative extension ratios are plotted in (B) as a function of the radial parameter in the swollen particle *r*, and shows both the radial and the orthogonal extension ratio normalized to the extension ratio *λ* for a homogeneous crosslinker distribution. The resulting density profile is shown as a function of *r* in (C) and corresponds to the full curve, normalized to the density *φ*_hom_ for the homogeneous distribution, which is the box profile corresponding to the dashed line. The analytical fit from eqn (7) using *R*/*R*_hom_ = 1.06, is shown by the circles, while the dotted line is the fuzzy-sphere model^51^ for *R*_c_/*R*_hom_ = 0.93 and *σ*/*R*_c_ = 0.15.

Different distributions of crosslinker throughout the particle^21^,^25^ can be considered with the method that we introduce above. Here, we choose the crosslinker profile from eqn (4) to solve eqn (6) and its boundary conditions numerically. This yields the mapping *r*_0_ → *r*(*r*_0_) between the collapsed and the swollen particle which is shown in Fig. 3A. The solid curve shows the projected coordinates in comparison with the homogeneously crosslinked network which is indicated by the straight dashed line. The heterogeneity results in a 6% larger radius of the swollen particle. From the calculated mapping we recover the local relative extension ratios of the network in both the radial direction as well as the orthogonal direction, which are shown in Fig. 3B. Close to the gel–water interface we observe large extensions in the radial direction, while the extension in the orthogonal directions remains approximately constant with respect to the core. This, therefore, demonstrates how the crosslinker gradient induces strong anisotropic swelling in the periphery of the particle. The corresponding gel density profile significantly deviates from a homogeneously crosslinked gel, as can be observed in Fig. 3C. We found that the density profile within the particle, 0 ≤ *r* ≤ *R*, can be accurately described by7
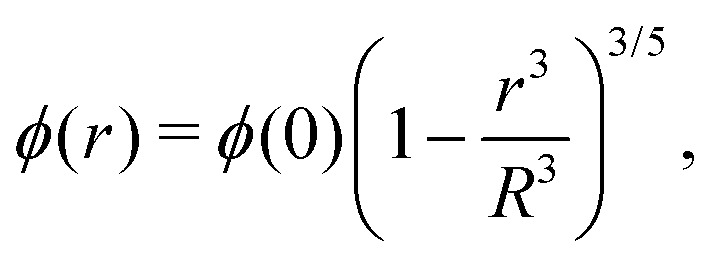
with *φ*(0) the core density that follows from the total amount of added polymer and *R* the radius of the particle. We proceed by comparing our approach to the fuzzy-sphere model,^51^ which results from convolution of the density profile of a sphere with a Gaussian distribution to account for the fuzzy nature of the polymer backbone. The dotted curve in Fig. 3C shows the latter density profile using a *σ* = 15% fuzziness. The core radius is chosen at *R*_c_ = 0.88*R* to align the first intensity minimum with the form factor resulting from eqn (7), as we will see below. Both density profiles deviate somewhat from each other, yet the most important conceptual difference is that eqn (7) yields a well-defined particle radius, while the density profile in fuzzy-sphere model gradually decays to zero.^51^

### 3.2 Comparison with SAXS data

Scattering studies yield a direct way to probe the gel network *in situ*, without the need for attaching (fluorescent) labels to the polymer backbone which could interact with the swelling or might be incorporated disproportional to the local gel density. An experimental study on the viscoelastic properties on microgels by Paloli and Crassous *et al.*^52^,^53^ includes small-angle x-ray scattering experiments (SAXS) on PNIPAM microgels with 7 wt% BIS as crosslinker. The scattering-intensity curves were measured for various temperatures. At low particle densities the single-particle scattering profiles are recovered and the intensity curve gives direct access to the form factor. Eqn (7) corresponds to a form factor8
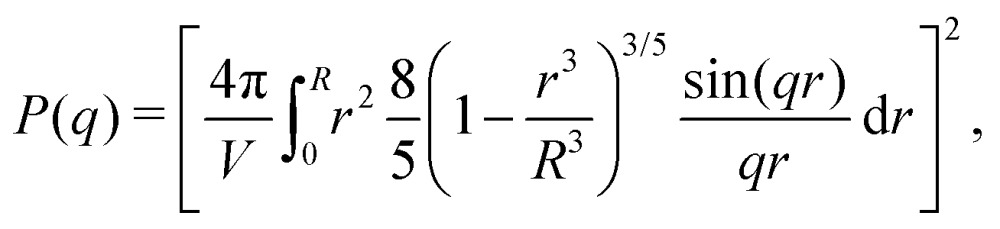
in which *q* is the magnitude of the scattering vector and *R* serves as the only fit parameter. The fuzzy-sphere model, on the other hand, uses9
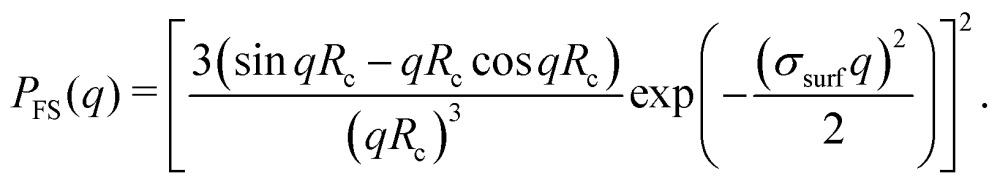
Here, *R*_c_ and *σ* are the core-radius and the fuzziness parameter which are the two fit parameters in the model.^51^ Due to the Gaussian distribution of mass, the polymer density profile in principle extends infinitely, but agreement with the measured hydrodynamic radius *R*_H_ has been reported by estimating *R*_H_ ≈ *R*_c_ + 2*σ*. The first minimum in eqn (8) appears at *qR* ≈ 5.12, contrary to *qR*_c_ ≈ 4.49 for a (fuzzy-) sphere model. An additional Lorentzian term *I*_fluct_(*q*) = *I*_0_/(1 + *ξ*^2^*q*^2^)^34^,^54^,^55^ is included in both models to take account for the network fluctuations, where *I*_0_ and *ξ* correspond to the scattering amplitude and mesh size of the polymer network that constitutes the microgel. The best fits with the data were obtained using *ξ* in the range 5–10 nm. No correction to account for experimental smearing was included, although this may improve further the correspondence between theory and experiments. At 31 °C we find *R* = 250 nm, and the fuzzy sphere fits *R*_c_ = 225 nm and *σ* = 30 nm. At 15 °C we fit *R* = 305 nm, and the fuzzy-sphere model was fitted using *R*_c_ = 268 and *σ* = 40 at this temperature. Both models properly capture the rapid decay of the intensity signal at higher *q*, compared to a simple sphere model. Eqn (8), however, fits more accurately the location on the higher-order minima, indicating improved accuracy of the underlying density profile.

### 3.3 Dangling ends

**Fig. 4:**
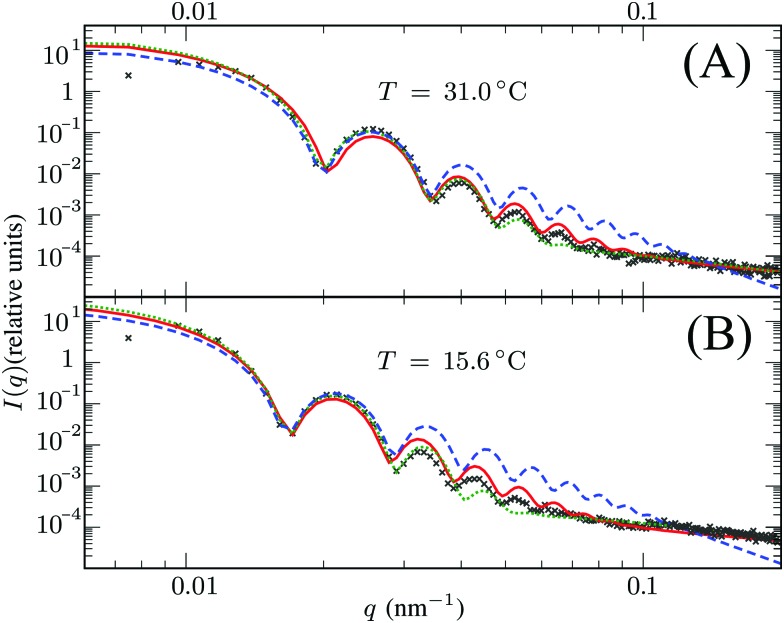
Intensity profiles for PNIPAM particles at (A) 31 °C and (B) 15 °C, showing the experimental data (symbols) and the model introduced in this work (solid red line) using *R* = 250 nm in (A) and *R* = 305 nm in (B). The green dotted line corresponds to the fuzzy sphere model, using *R* = 225 nm and *σ* = 30 nm in (A), and *R* = 268 nm and *σ* = 40 nm in (B). For completeness, we also show by the blue dashed line a simple spherical core with the same radius *R*_c_ as the fuzzy sphere. Both solid lines include an additional Lorentzian term to include a contribution from fluctuations of the network characterized by a mesh size *ξ*. The polydispersity was chosen 3.5–4% in all cases.

We speculate that the minor discrepancy between eqn (8) and the experimental data may result from the local density approximation which neglects the finite size of the polymer chains. Due to the relatively low crosslinker fraction the polymer chains that are close to the gel–water interface can have significant contour length and may either loop back to the network or terminate un-crosslinked. The expansive nature of these dangling ends, or dangling loops, may result in a be reflected in an additional increase of the hydrodynamic radius for temperatures below the VPTT, while for high temperatures the chains probably adsorb onto the collapsed core.^56^ For the system corresponding to Fig. 4, the hydrodynamic radius has been measured using dynamic light scattering at *R*_H_ = 300 ± 20 nm at 31 °C and *R*_H_ = 375 ± 20 nm at 15 °C, and is therefore considerably larger than the fitted radius using the density profile from eqn (7), in particular for temperatures far below the VPTT. This supports the hypothesis that the corona of dangling ends does not show up in static-light scattering or SAXS due to its low density, but affects the hydrodynamic properties of the particle nonetheless.^57^ The emergent picture is shown in Fig. 1, illustrating an inhomogeneously crosslinked network of radius *R* that undergoes non-uniform swelling as indicated by the calculated grid lines, combined with a corona of dangling ends that is much lower in density and contributes to the hydrodynamic radius *R*_H_.

In the ESI† we have included a brief study towards the properties of the dangling ends. Although more detailed studies will be needed, here we approximate the total number of dangling ends to be of the order of 10^4^ per particle, using parameters that match the experimental system. This yields a typical distance between grafting points *d* ≈ 10 nm. Most dangling ends seem to be fairly short and it is yet unclear whether a brush model^35^,^58^,^59^ can be applied to describe the repulsive interactions between the coronas or whether mushroom-like models^60^ might be more applicable instead. Future studies should elucidate the properties of the corona either from an experimental perspective, or from theoretical models or simulations which focus more closely on the crosslinking process. Those studies, therefore, could quantify the effect of the dangling ends on the hydrodynamic radius and the soft interaction between the microscopic gel particles.

## 4 Conclusion

Highly swollen hydrogels are characterized by simple scaling relations as well as a Poisson's ratio that reduces to 1/4, in agreement with Flory–Rehner theory. In this work we have obtained swelling profiles for microscopic PNIPAM hydrogel particles with an inhomogeneous crosslinker distribution. The calculated density profile corresponds to a scattering profile that matches well with experimental data after fitting the radius only. Interestingly, we observe a discrepancy between this radius and the measured hydrodynamic radius. This, we believe, points at the existence of a layer of dangling ends that extends considerably beyond the crosslinked core, and determines rheological and structural properties of microgel suspensions at low to moderate densities. The numerical method that we have introduced is versatile and can be extended towards charged networks by incorporation of the ionic osmotic pressure into the model.^61^ Charge inhomogeneities^62^ and also alternative distributions of cross linkers^25^ will be interesting research avenues for further research.

## Supplementary Material

Supplementary information

Supplementary information
