# Supplementary material for: Swelling of micro-hydrogels with a crosslinker gradient
Source: Phys Chem Chem Phys. 2017 Jun 6;19(35):23740–6. doi: 10.1039/c7cp02434g (PMC5778843; doi:10.1039/c7cp02434g)
Supplement: Supplementary file 2 [file CP-019-C7CP02434G-s002.pdf]

Electronic Supplementary Material (ESI) for Physical Chemistry Chemical Physics.
This journal is © the Owner Societies 2017

Supporting Information

In contrast to the general density profile that we derive in the main text, the grafting density and characteristic length of the dangling ends seem to depend non-trivially on the total number per particle of both the monomers as well as the crosslinker fraction. In this derivation we will, therefore, focus on experimental parameters corresponding to the SAXS data in Figure 4. Despite the relatively small contour length of the chains in the core region, the low crosslinker density close to the surface yields relatively long chains there. This is reflected in the *local* average number of monomers in a chain

$$M(r_0) = \frac{2M_0}{1-\frac{r_0^3}{R_0}}, \qquad (10)$$

which can be obtained from Eq. (4). Here, r_0 and R_0 the radial coordinate and particle radius in the collapsed particle. For the tetrafunctional network the global average number of monomers is $M_0 = N_m/2N_c$, where N_m and N_c are the number of monomers and the number of crosslinkers, respectively. From the experimental data we find $M_0 \approx 11$, and using the known density for collapsed PNIPAM gels[?] we find $N_c \approx 1.1 \cdot 10^8$ for a 200 nm particle at synthesis. We will assume the crosslinks are formed in a perfect melt, despite the geometrical limitations that the finite amount of polymer material in the growing particle imposes on the latter assumption. This yields a Gaussian distribution of end-to-end vectors originating from a radial coordinate r_0 in the collapsed particle,

$$P(\Delta r_0; r_0) = \sqrt{\frac{b^2 M(r_0)}{3M_k}} \exp\left(-\frac{3(\Delta r_0)^2 M_k}{2b^2 M(r_0)}\right) \qquad (11)$$

where $b \approx 2$ nm is the Kuhn length[?], and $M_k \approx 7.5$ is the associated number of monomers per Kuhn segment. We ignore curvature effects as we focus on the gel-water interface region for which $\Delta r_0 \ll r_0$, as we will show below. Furthermore, the statistical variation in the local segment size of the polymer chains has not been taken into account, yet we checked that this had only a minor effect on the results. Eq. (11) can be used to quantify the probability of a chain to reach the gel-water interface, and we apply the latter equation to approximate the fraction of polymer ends that remain un-crosslinked through the identification of end points that are located outside of the particle, i.e. $\Delta r_0 + r_0 > R_0$. This enables us to estimate a local mass fraction of dangling ends

$$f(r_0) = \frac{1}{2}\text{erfc}\left(-\frac{3(R_0 - r_0)^2 M_k}{2b^2 M(r_0)}\right). \qquad (12)$$

By integrating Eq. (12) over the interior of the (collapsed) particle we find that approximately 2% of the total mass of the particle remains dangling. We proceed by subtracting the corresponding local mass fractions from the polymer-network density profile that follows from Eq. (6), and redistribute this mass fraction by assuming that the dangling ends constitute a Gaussian density profile instead of confirming to the network. The same characteristic length as in the collapsed (melt) state Eq. (11) is used here. Note that this establishes a lower bound on the extension of the dangling ends here, as excluded volume interactions should yield an increased tendency for the chains to extend radially outwards into the solvent. The modified density profile is shown by the full curve in

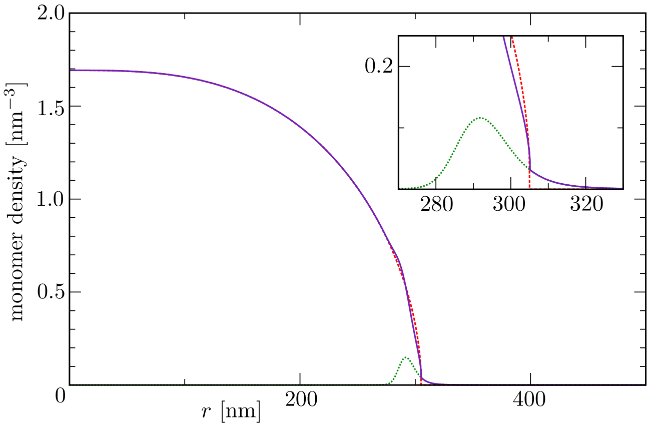

Fig. 5 Polymer-density profile for the swollen microgel with radius $R = 305$ nm. The red dashed curve corresponds to the density profile that was calculated in the main text and forms the base of our density profile. The green dotted curve corresponds to the calculated density of dangling ends (2% of total mass), resulting in a modified polymer-density profile that is shown by the purple solid curve. The inset shows a magnification of the region close to the gel-water interface.

Figure 5, and demonstrates a relatively small mass fraction originating from the dangling ends (green dotted curve) that extends beyond the boundary of the network. Because of the relatively small redistribution of mass the corresponding form factor was not affected and remains to coincide with Eq. (8) in this procedure. In

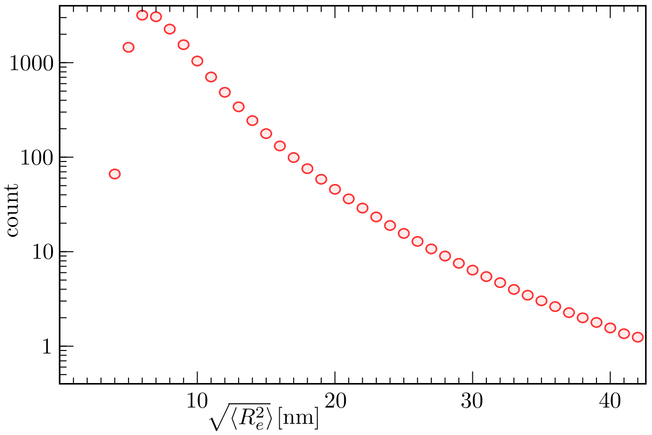

Fig. 6 Discretized distribution of end-to-end lengths of the dangling polymer chains. Here we use that $\langle R_e^2 \rangle = M(r_0)b^2/M_k$.

Figure 6 we plot the corresponding distribution of end-to-end distances of these dangling ends. We observe that the majority of the dangling ends is expected to extend only a short distance. A small minority is, however, capable of extending more than 30 nm into the solvent. It remains, however, for future research to determine the quantitative effect of these chains on the pair interactions as well as the hydrodynamic radius of the microgels.

References

S. Hirotsu, *J. Chem. Phys.*, 1991, **94**, 3949–3957.

K. Kubota, *Polym. J.*, 1990, **22**, 1051–1057.
